# Opinion limits study for the multi-selection bounded confidence model

**DOI:** 10.1371/journal.pone.0210745

**Published:** 2019-01-23

**Authors:** Jiangbo Zhang

**Affiliations:** School of science, Southwest Petroleum University; Chengdu 610500, P. R. China; Universidad de Burgos, SPAIN

## Abstract

In this work, we study the opinion limit states for a generalized bounded confidence agent-based opinion model. Agents can select multiple opinions in the network, and the confidence bound is considered on the distance between the average of the selected opinions and agent opinion itself. The number of selection agents for a certain agent, which is also called the selection number, means the agent opinion interaction degree. It is known that when the confidence bound is large sufficiently, opinions reach consensus almost surely. We mainly study the opinion consensus and the opinion polarization when the confidence bound is small sufficiently. Firstly, we provide and prove the upper and lower bounds for the opinion consensus probability of this bound confidence model. It shows that the opinion consensus probability almost always decreases as the confidence bound decreases. Secondly, the opinion consensus probability is larger than the one for the opinion evolution of the Deffuant-Weisbuch model. Finally, we demonstrate the ultimate probability distribution of one agent opinion and compare it with the gossip form and the general bounded confidence form, and demonstrate how the opinion polarization probabilities evolve as the selection number changes. Specially, different from other studies, we find that the opinion polarization would happen more easily if the opinion interaction degree is strengthened. In a sum, the multiple selection mechanism will increase the opinion consensus probability and the opinion polarization probability, respectively, comparing to the single selection mechanism.

## Introduction

### Background and motivation

How interactions among individual opinions promote to collective phenomena, is an important problem of social physics. Many social inspired models, such as the voter model, the majority rule model, the bounded confidence model and many other related agent-based models, are proposed and analyzed to understand the opinion formation mechanisms in various circumstances [1]. For the traditional graph model, social interactions are influenced heavily by the opinion communication graph modes, for example, opinions reach consensus among a fully connected graph and reach several limits in an unconnected network [2]. In decades, bounded confidence models were proposed, including the Hegselmann-Krause (HK) model [3] and the Deffuant-Weisbuch (DW) model [4]. The theoretical results [5] mainly show that the opinion fragmentation, a more complex social phenomenon, happens even in an underlying connected graph. This is corresponding to the real social network [6] such that agent opinion interactions not only lead to opinion consensus [7], but also opinion divergence [8]. These developments have received the attention of various scholars. Further studies on the bounded confidence models are generalized into the high dimensional condition [9], the continuous opinion case [10], the asymmetrical DW model case [11], the time-varying bounded confidence model [12], the noise-HK model case [13], and so on.

Opinion agreement had been studied widely for some bounded confidence models. For example, [13] proposed a noisy HK model and got that the noise promotes opinions gathering together even for the bounded confidence mechanism. [14] obtained that opinions reach consensus almost surely for a generalized DW model. From the viewpoint of the continuous opinion evolution, [10] discussed empirical opinion density using the system’s mean-field dynamics as the population size of the agents becomes sufficiently large. Recently, [15] proposed a bounded confidence and mobile opinion dynamics where opinions can reach stable under certain conditions. [16] studied the opinion models with certain logic constraints such as the French-DeGroot model, the linear Abelson model, the Friedkin-Johnsen model and the Taylor model, with the network methods to show how opinions evolve into agreement or disagreement. These literatures mainly got that a single class of opinion limits is analyzed for a single mechanism such as the mean-field dynamics, the influence dynamics and the bounded confidence dynamics [5].

In this paper, we will study a generalized DW model with the multiple phenomena evolution, where opinions will reach consensus with a positive probability and reach polarization with a positive probability, respectively. However, for most bounded confidence models [4, 17], the happening probability for each class of event still have not been measured. The reality example for the multiple phenomena evolution of individual opinion interactions includes the Wikipedia edit cooperation-conflict [18], such as different attitudes, approaches and emphases, where different opinion evolutions exist for one single social network. Thus, the probabilities of the opinion consensus event and the opinion polarization event should be measured for the further study of opinion dynamics [18]. Our research motivation is to study the opinion consensus and polarization phenomena for the generalized DW model when the confidence bound is sufficiently small. In fact, for bounded confidence models, opinions always reach several limits [4] and reach agreement when the confidence bound is sufficiently large. However, most results are obtained by computer simulations, such as the opinion convergence problem for the heterogeneous bounded confidence model [19]. In this paper, we will try to analyze the opinion evolution limit probabilities.

### Contribution and paper organization

In this paper, we will study and demonstrate the opinion fragmentation for a generalized DW model, the Long-range bounded confidence opinion model (LR model) [14] when the confidence bound is less than a certain threshold. Note that opinion consensus holds almost surely when the confidence bound is larger than a certain threshold [14]. Comparing to our previous works (see Table 1), this paper will study the multiple phenomena when the confidence bound is smaller than the threshold.

**Table 1:** Comparison between our work and some previous works.

*PhysicaA*2013	Opinion convergence study for the LMDW model and IMDW model
*JSSC*2015	The convergence rate estimations for the asymmetric DW model
*SIAM*2017	Fluctuation analysis for the Long-range (LR) model with the influence of stubborn agents
*Ours*	The opinion evolution analysis for the LR model when the confidence bound is small sufficiently

This work will be different from our previous works. Not only the parameter conditions, but also the probability methods that we will use are different.

Due to the confidence bound assigned to each regular agent of the LR model, this model is the state-dependent and highly nonlinear. Based on the independent selections, we mainly introduce the order statistics of the agent opinion sequence to estimate the consensus probability bounds for the LR model.

The main contributions in this paper include:

- We estimate the upper and lower bounds for the probability of opinion consensus for the LR model, when the confidence bound is small sufficiently.
- The relation of opinion consensus probability and the selection number for the LR model is analyzed. It shows that opinions will reach consensus with a larger probability for the multiple selection method comparing to the one for the single selection method.
- The opinion ultimate distributions for the LR model are demonstrated and are further compared to the gossip opinion model and the DW model with the same parameters. Besides, we also demonstrate and find the opinion polarization probability for the multiple selection mechanism will be larger than the one for the single selection mechanism.

The rest of this paper is organized as follows. First, we introduce preliminary background and formulates the opinion model, and then provides upper and lower bounds of agent opinion limit states’ consensus probability related with some parameters when the confidence bound is small sufficiently. Secondly, the probability comparison between the multiple selection and the single selection is provided. Furthermore, to shown the further opinion evolution phenomena, we demonstrate the final probability density of any agent opinion, comparing with the Gossip model and the Bounded-confidence model. Finally, the last section gives concluding remarks.

Notation: ℕ denotes the nonnegative integer number set {0, 1, 2, …}, while ℕ^+^ is the positive integer number set {1, 2, …}. ℝ is the real number set. ℝ^*m*×*n*^ means the *m* × *n* matrix with entries taking values in ℝ. **1**^*m*×*n*^ and **0**^*m*×*n*^ denote the *m* × *n* matrix with all entries being 1 and 0, respectively. dist(A,B)≜inf|b−a|:b∈A,a∈B is defined as the minimum distance of the set *A* and the set *B*. |*A*| is the interval length if *A* is a continuous interval and agent number if *A* is a discrete number set.

## Problem formulation

In this section, we consider an opinion dynamics with *n* agents and agent *i* takes an opinion real value in interval [0, 1] at time *t*, i∈V={1,…,n}, *t* ∈ ℕ. Different from the traditional bounded confidence model, the key features of our model are: 1) its bounded confidence restrictions for average opinions (the acceptable degree of the selected average opinions); 2) the multi-selection long-range learning [20].

### Model description

It is time to give a mathematical description of our opinion model. The evolution of *x*_*i*_(*t*) (any agent *i*’s opinion at time *t*) is given by

$$\begin{array}{c} \left\{ \begin{array}{cc} & x_{i}\left(t+1\right)=x_{i}\left(t\right)+\delta 1_{\left\{|y_{i}\left(t\right)|\leq \varepsilon_{0}\right\}}\cdot y_{i}\left(t\right),\\ & y_{i}\left(t\right)=\frac{\sum_{j=1}^{c}x_{r_{i}^{\left(j\right)}\left(t\right)}\left(t\right)}{c}-x_{i}\left(t\right), \end{array}\right. \end{array}$$ (1)

for i∈V, *t* ≥ 0, where *x*_*i*_(0) is selected uniformly in [0, 1] and

- $r_{i}^{\left(j\right)}\left(t\right)$ denotes the agent index selected by agent *i* at its *j*-th selection for the time *t* ∈ ℕ, distributed uniformly in V.
- *c* is the finite positive integer number of agents selected by an agent in V (here we assume that any agent in V can be selected repeatedly);
- the confidence bound *ε*_0_ ∈ (0, 1) represents the opinion confidential range radius ([1]);
- *δ* ∈ (0, 1) is the combination weight;
- *y*_*i*_(*t*) is the difference between the average opinion and the opinion of agent *i* itself.

For this model, the probability space can be denoted as (Ω,F,P), where Ω is a set composed by the Decare product of all possible initial states and all possible selections, and **P** is a normalized measure on a *σ*-algebra F of subsets of Ω (referring to [21]). $\left\{F_{t}\right\}$ is a filtration on the *σ*-algebra F. $1_{\left[\text{inequality}\right]}$ is the indicator function: $1_{\left[\text{inequality}\right]}=1$ if the inequality is fulfilled, and $1_{\left[\text{inequality}\right]}=0$ otherwise ([22]).

**Remark 1** *In our model, if the weighted average is in its opinion confidence, then the agent updates its opinion with the weighted opinion average of agents selected randomly in* V.

*Also, this agent opinion can be influenced by some opinions out of its own confidence range* $R_{i}\left(t\right)$, *which stands for a “long-range” rule. Moreover, because of the confidence bound ε*_0_, *the indicator function* $1_{\left\{|y_{i}\left(t\right)|\leq \varepsilon_{0}\right\}}$ *depends on opinion states x_i_*(*t*), i∈V, *and therefore, the model is state-dependent*.

### Basic definitions

First we provide some definitions on the probability of opinion evolution.

**Definition 1** *We define the opinion orders as follows*:

- *Denote x*_[*i*]_(*t*) *as the i-th agent opinion value at time t satisfying x*_[1]_(*t*) ≤ *x*_[2]_(*t*) ≤ ⋯ ≤ *x*_[*n*]_(*t*).
- *The i-th consecutive difference of the order statistics x*_[*i*+1]_(*t*) *and x*_[*i*]_(*t*) *is denoted as D*_[*i*,*i*+1]_(*t*) = *x*_[*i*+1]_(*t*) − *x*_[*i*]_(*t*)*; Specially, the opinion range at time t is* Δ(*t*) = *x*_[*n*]_(*t*) − *x*_[1]_(*t*).

**Remark 2** *Apparently reaching a consensus of model* (1) *implies that* lim_*t*→∞_ Δ(*t*) = 0. *Besides, for the model* (1), Δ(*t*) *is decreasing. By the model* (1), *the opinion range* Δ(*t*) *will decrease as t grows*.

## Opinion consensus estimation

In this section, we present the main results of the model (1). Concretely, we provide the bound estimations of the opinion consensus probability and study the influence of the selection number *c* on the opinion consensus probability.

### Main results

We present the following three main results, respectively.

**Theorem 1** *For the LR model* (1), *if* $\varepsilon_{0}<\frac{1}{c}$, *then*

$$\begin{array}{c} P\left(\underset{t\to \infty }{\text{lim}}\Delta \left(t\right)=0\right)\geq n{\left(c\varepsilon_{0}\right)}^{n-1}-\left(n-1\right){\left(c\varepsilon_{0}\right)}^{n}. \end{array}$$

**Remark 3** *For the lower probability bound of opinion consensus, this bound is larger than 0, which can be obtained by n* > *n* − 1 *and* (*cε*_0_)^*n*−1^ > (*cε*_0_)^*n*^ *obviously*.

**Theorem 2** *For the LR model* (1), *if* $\varepsilon_{0}<\frac{1}{c}$, *then*

$$\begin{array}{c} P\left(\underset{t\to \infty }{\text{lim}}\Delta \left(t\right)=0\right)\leq \{\begin{array}{cc} n!\;(c\varepsilon_{0}{)}^{n-1}, & if\;n\leq \lceil \frac{1}{c\varepsilon_{0}}\rceil \\ n!\;(c\varepsilon_{0}{)}^{n-\lfloor \frac{1}{c\varepsilon_{0}}\rfloor }, & otherwise. \end{array} \end{array}$$

**Remark 4** *For the upper probability bound of opinion consensus, the bounds are smaller than 1 according to the Stirling’s approximation. In fact*, $n!\leq e\sqrt{n}{\left(\frac{n}{e}\right)}^{n}$, *thus*

$$\begin{array}{c} n!(c\varepsilon_{0}{)}^{n-1}\leq n!{\left(\frac{1}{n}\right)}^{n-1}\leq \frac{n\sqrt{n}}{e^{n-1}}<1\;for\;any\;n\geq 3 \end{array}$$

*and*

$$\begin{array}{c} n!\;(c\varepsilon_{0}{)}^{n-\lfloor \frac{1}{c\varepsilon_{0}}\rfloor }\leq n!\;(\frac{1}{n}{)}^{n-\lfloor \frac{1}{c\varepsilon_{0}}\rfloor }\leq \frac{n^{\left\lfloor \frac{1}{c\varepsilon_{0}}\right\rfloor }\sqrt{n}}{e^{n-1}}<1\;for\;any\;n\geq \left(\left\lceil \frac{1}{c\varepsilon_{0}}\right\rceil +\frac{1}{2}\right)\;\text{ln}\;\left(\left\lceil \frac{1}{c\varepsilon_{0}}\right\rceil +\frac{1}{2}\right). \end{array}$$

For the opinion model (1), we can denote Δ(*t*) further into Δ_*c*_(*t*) where *c* is the selection number and *c* > 1.

**Theorem 3** *If* $\varepsilon_{0}<\frac{1}{cn}$, *n* > 2 *and* $c>\sqrt[{n-1}]{\frac{n!}{n-1}}$, *then*

$$\begin{array}{c} P\left(\underset{t\to \infty }{\text{lim}}\Delta_{c}\left(t\right)=0\right)>P\left(\underset{t\to \infty }{\text{lim}}\Delta_{1}\left(t\right)=0\right). \end{array}$$ (2)

**Remark 5** *We remark that* $\sqrt[{n-1}]{\frac{n!}{n-1}}<n$ *or* *n*! < (*n* − 1)*n*^*n*−1^ *when n* ≥ 3 *by the String’s approximation. In fact*, $n!<e\sqrt{n}{\left(\frac{n}{e}\right)}^{n}$. *Thus, to obtain* $e\sqrt{n}{\left(\frac{n}{e}\right)}^{n}<\left(n-1\right)n^{n-1}$, *we get that* $n\sqrt{n}<\left(n-1\right)e^{n-1}$ *which holds obviously when n* ≥ 3. *If n* = 2, *then* $\sqrt[{n-1}]{\frac{n!}{n-1}}=2=n$.

*Specially, this theorem shows the multiple selection rule can promote the opinion consensus comparing to the single selection rule. Furthermore, we will demonstrate the opinion polarization phenomena and show that the multiple selection rule can also increase the opinion polarization probability*.

All the supporting Lemmas and proofs for the three Theorems are provided in Appendix.

## Simulations for the opinion distribution

In this section, we will first simulate the opinion consensus probability to illustrate how opinion consensus probabilities change, and then compare the opinion limit distributions among the Gossip model, the LR model and the bounded-confidence model. Finally, we demonstrate the opinion polarization probabilities.

### Opinion Consensus probability bounds

**Fig 1:**
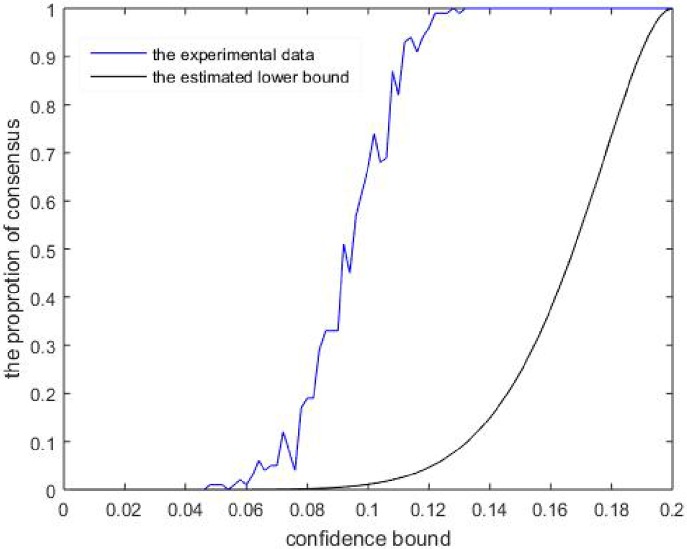
The probability of opinion consensus as *ε*_0_ increases. The estimated curve is calculated by the proportion of Δ(*T*) < *ε*_0_ for 100 times of simulations.

Theorems 1 and 2 reflect that the probability of opinion consensus when $\varepsilon_{0}<\frac{1}{c}$ can be bounded by some parameters. We now simulate the opinion consensus probability in Fig 1.

In this section, simulations were performed using the software MATLAB R2015a. Set the regular agent number *n* = 10, the termination time *T* = 10^5^, the selection number *c* = 5 and *δ* = 0.4. In the simulation, we use Δ(*T*) < *ε*_0_ to denote the opinion consensus because agent opinions must reach consensus if Δ(*t*) < *ε*_0_ for a certain finite *t* ∈ ℕ. Fig 1 compares the lower estimated opinion consensus probability with the estimated opinion consensus probability. Note that the estimated curve will take downwards when the simulation termination time increases, thus we only compare the analytical lower bound but not the analytical upper bound. The upper bound will be larger than the values of estimated curve when the termination time is large enough but hard to process.

### Probability distributions

As pointed out in previous discussions, by analyzing **P**(lim_*t*→∞_ |*x*_*i*_(*t*) − *x*_*j*_(*t*)| > *cε*_0_), *i* ≠ *j*, we can estimate the consensus probability. However, finding or estimating the accurate opinion consensus probability by the mathematical methods is not scalable.

Based on the model (1), it is straightforward to see that the limit distribution for all agent opinions are the same. Thus, if we can obtain the limit distribution for any one agent opinion, the exact probability for any limit numbers can be obtained. It is nearly impossible to be solved by the mathematical method. Thus, here we demonstrate the limited distribution for any agent opinion for the LR model.

To show the difference from other models, we can get a traditional gossip model for *c* = 1 and *ε*_0_ = 1; while we can get a traditional bounded-confidence model for *c* = 1. Now we can see the probability densities of limit opinion states for these three models as follows, where *n* = 30, *δ* = 0.5, *c* = 4 and the terminal time *T* = 10^5^.

**Fig 2:**
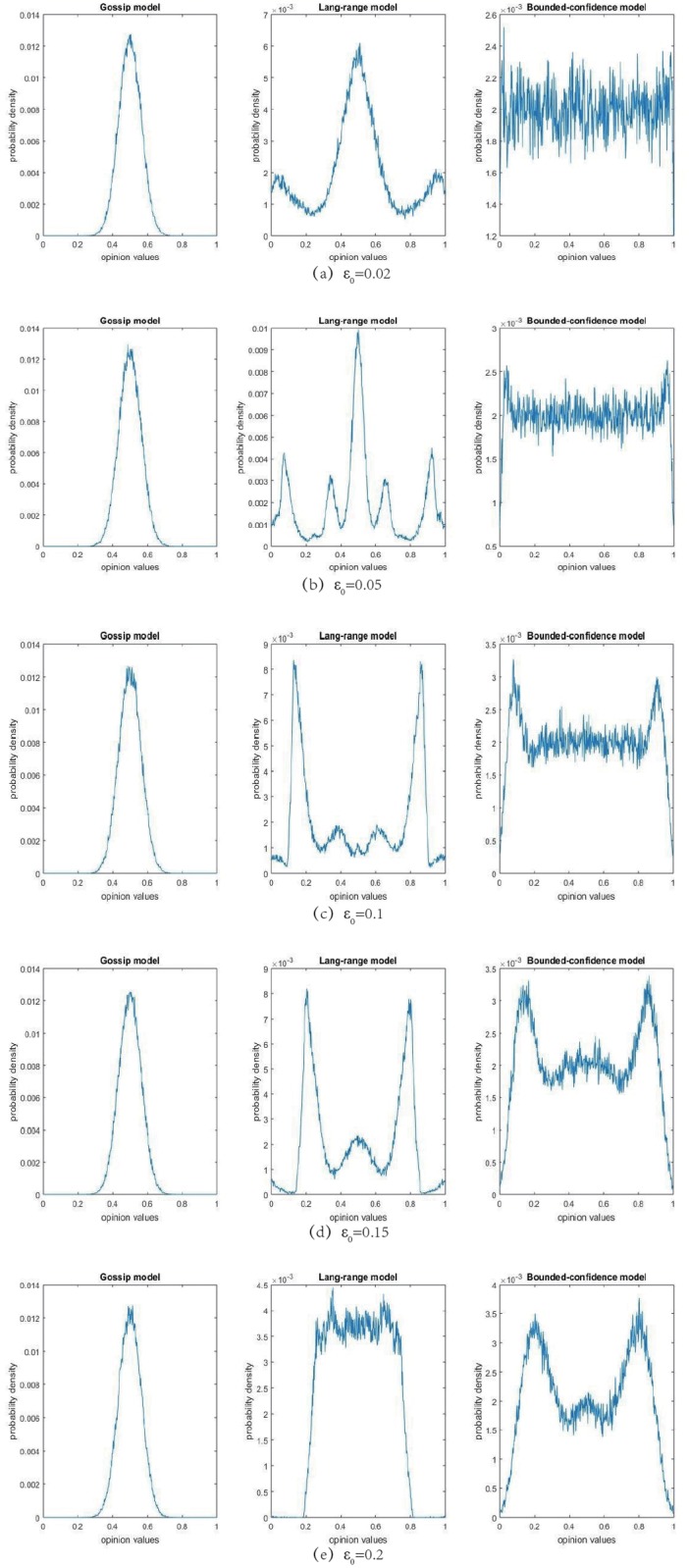
The change of one single agent opinion’s limit distribution.

All the simulations are the average of 100 different repetitions. By Theorem 3, when $\varepsilon_{0}<\frac{1}{cn}$, *n* > 2 and $c>\sqrt[{n-1}]{\frac{n!}{n-1}}$, the multiple selection rule can promote the opinion consensus comparing to the single selection rule. In fact, the most matched graph is Fig 2(a), by which we can find that opinions are more easily to be aggregated in the middle part comparing to the bounded-confidence model. As the confidence bound becomes larger, the opinion evolutions are more complex due to the conditions of Theorem 3 are not satisfied.

From Fig 2, we can get the following enlightenments:

1. When the confidence bound is sufficiently small (*ε*_0_ = 0.02), it is appeared that the limit distribution for the LR model is more nearly with the one for the Gossip model.
2. Comparing with Fig 2(a), the fragmentation of the opinion limit peaks appears more in this figure (*ε*_0_ = 0.05).
3. As shown in this figure (*ε*_0_ = 0.1), the polarization phenomenon appears more obvious than Fig 2(b). In fact, note that the condition of this figure is much different from the one of Theorem 3, thus the the multiple selection rule can increase the opinion polarization probability when *ε*_0_ is large enough but can also increase the opinion consensus probability when *ε*_0_ is small enough.
4. Comparing with Figs 2(c) and 1(d) shows that the two peaks approach to each other and there appears a peak in the middle when *ε*_0_ = 0.15.
5. When *ε*_0_ = 0.2, the aggregated effect into the middle part is enhanced. As shown in [14], when $\varepsilon_{0}>\frac{1}{c}$, these three opinion models will have the same opinion final distributions.

In a summery, it is shown that the opinion polarization appears in the limit distribution of the LR model but is not obvious for another two models. In the following subsection, we will demonstrate the polarization probability for the LR model.

### Polarization probability

Fig 2(c) and 2(d) raise a problem. Does the multi-selection mechanism increases the polarization probability of opinion convergence?

In this part, we provide the following simulation, which shows that as the selection number *c* increases, the polarization probability would also increase. In this simulation, we calculate the average of 100 simulations where the polarization probabilities are estimated by the frequency of Δ(*T*) − *D*_[*i*,*i*+1]_(*T*) < *ε*_0_ and *D*_[*i*,*i*+1]_(*T*) > *ε*_0_ for certain i∈V. In fact, from the graph theory viewpoint, the more the selection number *c*, the more frequent the random edge connections. Thus, opinions will aggregate into few numbers of clusters.

**Fig 3:**
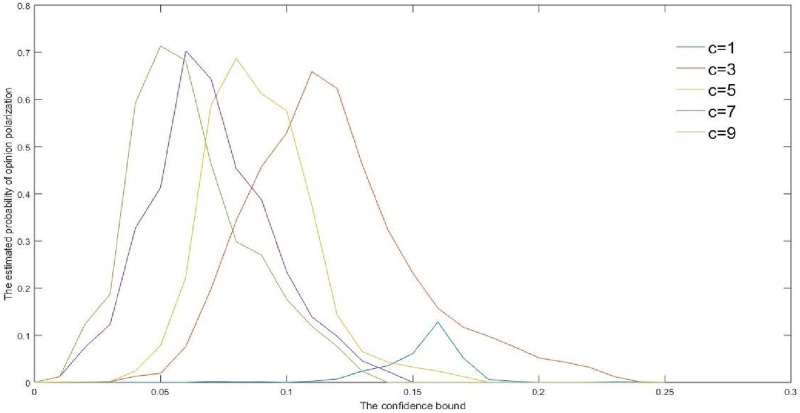
The probability of opinion polarization as the confidence bound increases.

For Fig 3, we can get the following enlightenments:

1. When the selection number *c* is large enough and the confidence bound is less than a certain value, the probability of the opinion polarization is larger than the condition for *c* = 1 markedly.
2. [23] promotes the necessity of the prevention for the opinion polarization. While Fig 3 illustrates that the opinion polarization occurrence probability can be reduced by cutting off the opinion multi-selection links, if the opinion confidence bound is sufficiently small.
3. Note that this result is really different from the conclusion shown in [24] that the opinion interaction can strengthen the social network connection and make opinions reaching agreements more easily. A possible explanation is that if the confidence bound is small enough, opinion communications would be strengthened in the separated subgroups but not among the whole group. In fact, this can reflect some real social phenomena [23].

## Conclusions

In this paper, we study the LR model when $\varepsilon_{0}<\frac{1}{c}$, whose opinion evolutions are more diversified than the condition for $\varepsilon_{0}\geq \frac{1}{c}$. First, we provided upper and lower bounds of opinion consensus related with the parameters *n*, *c* and *ε*_0_. Then, if *c* is large enough, then we studied the relation of opinion consensus probability and the consensus probability for *c* = 1. Finally, simulations for the opinion consensus probability, opinion final distributions and the relation of the opinion polarization and the selection numbers are invested. These analysis and simulations help us to understand how opinions evolve for the LR model when the confidence bound is small sufficiently. However, the opinion polarization probability for the multiple selection number is larger than the one for the single selection number.

There must be some further opinion evolution problems for the bounded confidence model that we can study. For some other models, many interesting and challenging problems on the opinion evolution phenomena of the social networks can be found and remain to be done. Thus, the study of some composite models with certain social backgrounds will be paid more attentions.

## Appendix

### Supporting lemmas

We first provide the following lemma, whose proof is provided in [25].

**Lemma 1** *The probability distribution of D*_[*i*,*i*+1]_(0) *obeys β*(1, *n*), 1 ≤ *i* ≤ *n* − 1, *where its probability density is*

$$\begin{array}{c} p\left(x\right)=\{\begin{array}{ll} \frac{\Gamma \left(1+n\right)}{\Gamma \left(1\right)\Gamma \left(n\right)}{\left(1-x\right)}^{n-1} & \;,0<x<1\\ 0 & ,others. \end{array} \end{array}$$

Specially, the probability distribution of Δ(0) obeys *β*(*n* − 1, 2).

**Remark 6** *The proof of this lemma can be obtained easily by studying the difference of the uniform distribution’s order statistics. For the reason of state-dependent evolution of the model* 1, *it is hard to provide the distribution of D*_[*i*,*i*+1]_(*t*) *for any t* ≥ 1. *However, at least we can find the following special condition. Besides, we can obtain the joint probability density of the order statistics* (*D*_[1,2]_(0), *D*_[2,3]_, …, *D*_[*n*−1, *n*]_(0)) *as shown in the following lemma*.

By Theorem 3.1 in Chapter 4.3 in [26], we can get that

**Lemma 2** *For the joint distribution of the consecutive order statistics*

$$\begin{array}{c} (x_{\left[1\right]}\left(0\right),D_{\left[1,2\right]}\left(0\right),D_{\left[2,3\right]},\ldots ,D_{\left[n-1,n\right]}\left(0\right)), \end{array}$$

we obtain its density as follows

$$\begin{array}{c} f_{\left(x_{\left[1\right]}\left(0\right),D_{\left[1,2\right]}\left(0\right),D_{\left[2,3\right]},\ldots ,D_{\left[n-1,n\right]}\left(0\right)\right)}\left(x_{1},x_{2},\ldots ,x_{n}\right)=n!,0<x_{k}<1,\sum_{k=1}^{n}x_{k}\leq 1,1\leq k\leq n. \end{array}$$

In the following, we will provide the condition that agent opinions converge into 2 limits.

**Lemma 3** *For any η* > 0, *if D*_[*i*, *i*+1]_(*t*) > (*c* + *η*)*ε*_0_ *and* $\text{max}\left\{D_{\left[1,i\right]}\left(t\right),D_{\left[i+1,n\right]}\left(t\right)\right\}<\frac{\eta }{c-1}\varepsilon_{0}$ *for certain* i∈V *and certain moment t* ∈ ℕ, *then agent opinions will converge into 2 limit states*.

**Proof**: Denote the agent opinion groups *S*_1_ = {1, 2, …, *i*} and *S*_2_ = {*i* + 1, *i* + 2, …, *n*}. Without loss of generality, we consider the time 0. For any agent *k* ∈ *S*_1_, it is not difficult to prove that one of the “slowest” selection methods for it moving into another group *S*_2_ is that $r_{k}^{\left(1\right)}\left(0\right)=\left[i+1\right]$ and $r_{k}^{\left(j\right)}\left(0\right)=\left[1\right]$, *j* = 2, …, *c* [14]. However,

$$\begin{array}{c} |y_{k}\left(0\right)|=|\frac{1}{c}x_{r_{k}^{\left(1\right)}\left(0\right)}\left(0\right)+\frac{1}{c}\sum_{j\neq 1}x_{r_{k}^{\left(j\right)}\left(0\right)}\left(0\right)-x_{k}\left(0\right)|\\ =\frac{1}{c}\left(x_{\left[i+1\right]}\left(0\right)-x_{k}\left(0\right)\right)-\frac{c-1}{c}\left(x_{k}\left(0\right)-x_{\left[1\right]}\left(0\right)\right)>\frac{1}{c}\left(c+\eta \right)\varepsilon_{0}-\frac{c-1}{c}\frac{\eta }{c-1}\varepsilon_{0}=\varepsilon_{0}. \end{array}$$

Symmetrically, we can get a similar result for the agent index in *S*_2_. Thus, for any selections, agents in *S*_1_ and *S*_2_ can not merge together. The conclusion is obtained.

**Remark 7** *The result of this lemma shows that agent opinion groups will not merge if their distances are large enough. However, it is not easy to generate into 3 limits or more. Because any agent i’s selection method could balance the difference y*_*i*_(0) *to make* |*y*_*i*_(0)| < *ε*_0_, *by selecting one agent from the group in the left direction and another agent from the group in the right direction*.

**Lemma 4** *For the model* (1), *opinions will convergence a.s. for any ε*_0_ ∈ (0, 1) *and c* ∈ ℕ. *Besides, for any* i,j∈V,

$$\begin{array}{c} \underset{t\to \infty }{\text{lim}}|x_{i}\left(t\right)-x_{j}\left(t\right)|=0\;or\;\underset{t\to \infty }{\text{lim}}\left|x_{i}\left(t\right)-x_{j}\left(t\right)\right|>c\varepsilon_{0}. \end{array}$$

**Remark 8** *This lemma can be proved with the infinite flow stability method used in* [14]. *The conclusion is similar with the convergence of the SMDW model in* [14], *while the difference is Lemma 4 shows agent opinions will converge with a larger distance compare with the SMDW model. This can be obtained by the contradiction method used in* [27].

### Proof of Theorem 1

Through the decreasing of {Δ(*t*)},

$$\begin{array}{c} P\left(\underset{t\to \infty }{\text{lim}}\Delta \left(t\right)=0\right)\geq P\left(\Delta \left(0\right)<c\varepsilon_{0}\right). \end{array}$$

By Lemma 1,

$$\begin{array}{c} P\left(\Delta \left(0\right)<c\varepsilon_{0}\right)=\int_{0}^{c\varepsilon }\frac{\Gamma (n+1)}{\Gamma (n-1)\Gamma (2)}x^{n-2}\left(1-x\right)dx\\ =n\left(n-1\right)\int_{0}^{c\varepsilon_{0}}x^{n-2}\left(1-x\right)dx\\ =n{\left(c\varepsilon_{0}\right)}^{n-1}-\left(n-1\right){\left(c\varepsilon_{0}\right)}^{n}. \end{array}$$

The conclusion follows.

By this theorem, we initially obtain the relationship of the opinion consensus’s lower probability bound and the agent parameters.

### Proof of Theorem 2

Similar with Lemma 3, we can get that **P**(lim_*t*→∞_ Δ(*t*) = 0) ≤ 1 − **P**(*A*), where $A=\{D_{\left[i,i+1\right]}\left(0\right)>c\varepsilon_{0},\exists i\in V\}$. Note that the number of the agent *i* such that *D*_[*i*,*i*+1]_(0) > *cε*_0_ satisfies $|\left\{i:D_{\left[i,i+1\right]}\left(0\right)>c\varepsilon_{0}\right\}|<\left\lfloor \frac{1}{c\varepsilon_{0}}\right\rfloor$. By the Lemma 2, if $n\leq \lfloor \frac{1}{c\varepsilon_{0}}\rfloor +1$, then we can obtain that

$$\begin{array}{cc} P(A) & =\int_{\exists k\in V,D_{\left[k,k+1\right]}\left(0\right)>c\varepsilon_{0}}n!dx_{1}\ldots dx_{n}\\ & =1-\int_{\forall k\in V,D_{\left[k,k+1\right]}\left(0\right)\leq c\varepsilon_{0}}n!dx_{1}\ldots dx_{n}\\ & =1-n!\int_{0}^{1}dx_{1}\int_{0}^{c\varepsilon_{0}}\underset{n}{\underset{︸}{\ldots }}\int_{0}^{c\varepsilon_{0}}dx_{2}dx_{3}\ldots dx_{n}\\ & =1-n!\;(c\varepsilon_{0}{)}^{n-1}. \end{array}$$

Thus, if $n\leq \lfloor \frac{1}{c\varepsilon_{0}}\rfloor +1$, then

$$\begin{array}{c} P\left(\underset{t\to \infty }{\text{lim}}\Delta \left(t\right)=0\right)\leq 1-P\left(A\right)\leq 1-1+n!\;(c\varepsilon_{0}{)}^{n-1}=n!\;(c\varepsilon_{0}{)}^{n-1}. \end{array}$$

While when $n>\lfloor \frac{1}{c\varepsilon_{0}}\rfloor +1$,

$$\begin{array}{c} P\left(A\right)\leq 1-n!\int_{0}^{1}dx_{1}\int_{0}^{c\varepsilon_{0}}\underset{n-\lfloor \frac{1}{c\varepsilon_{0}}\rfloor }{\underset{︸}{\ldots }}\int_{0}^{c\varepsilon_{0}}dx_{2}dx_{3}\ldots dx_{n}\geq 1-n!\;(c\varepsilon_{0}{)}^{n-\lfloor \frac{1}{c\varepsilon_{0}}\rfloor }. \end{array}$$

Thus, if $n>\lfloor \frac{1}{c\varepsilon_{0}}\rfloor +1$, then

$$\begin{array}{c} P\left(\underset{t\to \infty }{\text{lim}}\Delta \left(t\right)=0\right)\leq 1-P\left(A\right)\leq n!\;(c\varepsilon_{0}{)}^{n-\lfloor \frac{1}{c\varepsilon_{0}}\rfloor }. \end{array}$$

The conclusion follows.

### Proof of Theorem 3

To compare the consensus effort of agent multi-selection and traditional single-selection (that is, the asymmetrical DW model), we can deduce that when *c* is larger enough, the agent consensus probability of the LR model are more larger than the one of the asymmetrical DW model, which can be stated in the following corollary.

By Lemma 4 and Theorem 2, we get that if

$$\begin{array}{c} n{\left(c\varepsilon_{0}\right)}^{n-1}-\left(n-1\right){\left(c\varepsilon_{0}\right)}^{n}>n!\varepsilon_{0}^{n-1}, \end{array}$$ (3)

where $\varepsilon_{0}<\frac{1}{cn}$, then the conclusion (2) follows.

Furthermore, note that

$$\begin{array}{c} (n-\frac{n-1}{n})c^{n-1}>\left(n-1\right)c^{n-1}>n! \end{array}$$

can induce

$$\begin{array}{c} nc^{n-1}-\left(n-1\right)c^{n}\varepsilon_{0}>nc^{n-1}-\left(n-1\right)c^{n}\frac{1}{cn}>n!. \end{array}$$

Both sides are multiplied by $\varepsilon_{0}^{n-1}$, then we obtain the inequality (3). Thus the conclusion follows.

**Remark 9** *Theorem 3 shows that when c is large enough, the consensus probability for the multi-selection mechanism is larger than the one for the single-selection mechanism. Note that this is obtained by the theoretical analysis*.

## Supporting information

S1 File LRdensity.m. This m-file is to demonstrate the opinion density for the LR model.
(ZIP)

S2 File LRdynamic.m. This m-file is to simulation the LR model and judge whether opinions reach polarization at the termination time.
(ZIP)

S3 File Polarize.m. This m-file is to estimate the proportion of the polarization events given one certain parameters.
(ZIP)

S4 File Polgraph.m. This m-file is to estimate the proportions of the polarization events comparing different parameters.
(ZIP)

S5 File rateLRdynamic.m. This m-file calculates the proportion of the polarization events.
(ZIP)

S6 File tuberbound.m. This m-file calculates the opinion range at the termination time for the LR model.
(ZIP)
